# Supplementary material for: Motion prediction enables simulated MR-imaging of freely moving model organisms
Source: PLoS Comput Biol. 2019 Dec 19;15(12):e1006997. doi: 10.1371/journal.pcbi.1006997 (PMC6941817; doi:10.1371/journal.pcbi.1006997)
Supplement: S1 Text — (PDF) [file pcbi.1006997.s001.pdf]

Supporting information for: Motion prediction enables simulated MR-imaging of freely moving model organisms

Markus Reischl[1◑], Mazin Jouda[2◑], Neil MacKinnon[2], Erwin Fuhrer[2], Natalia Bakhtina[2¤], Andreas Bartschat[1], Ralf Mikut[1], Jan G. Korvink[2*]

1 Institute for Automation and Applied Informatics, Karlsruhe Institute of Technology, Hermann-von-Helmholtz-Platz 1, 76344 Eggenstein-Leopoldshafen, Germany
2 Institute of Microstructure Technology, Karlsruhe Institute of Technology, Hermann-von-Helmholtz-Platz 1, 76344 Eggenstein-Leopoldshafen, Germany

* jan.korvink@kit.edu

Supporting information

Motion prediction

This section describes the principal structure of the algorithm. Free parameters are chosen heuristically for the given dataset. Raw videos are streams of RGB images with m rows and n columns at each frame $k = 1, ..., K$. Indices i, j denote x- and y-position in the image, and index r denotes the color:

$$\mathbf{X}^{\text{raw}} = (x_{i,j,r}^{\text{raw}}[k]), \quad x_{i,j,r}^{\text{raw}}[k] \in \{0, ..., 255\}. \tag{1}$$

We use a standard MATLAB conversion to convert the images to grayscale and keep only $1/z^2$ of all pixels to speed up subsequent performance (every z-th line, every z-th column is kept, here $z = 3$, see Fig 3):

$$x_{i,j}[k] = \sum_{r=1}^{3} c_r \cdot x_{z\cdot i, z\cdot j, r}^{\text{raw}}[k], i = 1, ..., \frac{m}{z}, j = 1, ..., \frac{n}{z}, \tag{2}$$

$$\text{here} : c_1 = 0.2989, c_2 = 0.5870, c_3 = 0.1140. \tag{3}$$

To segment the worm, a simple threshold segmentation will be erroneous due to illumination effects, noise, and dark corners. To be independent of illumination effects, we generate a background image excluding the worm by calculating at each pixel position the 75th percentile[1] along the time scale $\overline{\mathbf{X}}^{0.75} = (\overline{x}_{i,j}^{0.75})$. The obtained background estimation is smoothed by a closing step (structuring element $\mathbf{B}$: DISC, $r = 3$):

$$\mathbf{X}^{\text{back}} = (\overline{\mathbf{X}}^{0.75} \oplus \mathbf{B}) \ominus \mathbf{B} \tag{4}$$

where the operators $\oplus$ and $\ominus$ respectively denote the dilation and erosion. To obtain the worm segment, we subtract the background estimation from each image frame (Fig 3d).

As a threshold for the segmentation of the worm, we choose the 1st percentile $\overline{x}_k^{0.01}$ of all $m \cdot n$ pixels in the difference image at every time step (assuming that the worm covers an area of less than 1% of the image). We then discard all remaining objects except the biggest one. As an outcome, we obtain image frames $\mathbf{X}^{\text{worm}}[k]$ of the segmented worm.

[1]The value delivers the brightness of the background pixel, if the worm does not occupy a pixel for more than 25% of the time.

Within the worm segment, the direction of the worm needs to be identified. This can be done by estimating the position of the head and the center of gravity (COG). We calculate for each time sample k the COG based on the pixels in the binary image $\mathbf{X}^{\text{worm}}[k]$. The found values $\mathbf{x}^{\text{raw}}_{\text{COG}}[k] = (x^{\text{raw}}_{1,\text{COG}}[k], x^{\text{raw}}_{2,\text{COG}}[k])^T$ are then smoothed (1st order low-pass filtering over time) to obtain variations for future predictions. Due to quantization sometimes the COG does not move, therefore prediction algorithms might fail. The resulting time series is denoted $\mathbf{x}_{\text{COG}}[k] = (x_{1,\text{COG}}[k], x_{2,\text{COG}}[k])^T$.

To find the head, the moving parts of the worm need to be identified. Observations show that the head is always moving left/right, the tail does not. We calculate an optical flow image which contains the difference of two succeeding segmentations

$$\mathbf{X}^{\text{mov}}[k] = \mathbf{X}^{\text{worm}}[k] - \mathbf{X}^{\text{worm}}[k-1]. \tag{5}$$

We use the integer values to distinguish between pixels where the worm moved to (value 1), and those, where it came from (value -1). For each time sample, we then sum up the last 10 optical flow images $\mathbf{X}^{\text{mov}}[k]$ (this could also be done by simply subtracting images with $\Delta k = 10$, discard all negative values (body moved away), apply an opening (=denoising and deletion of pixels along the complete body). The resulting values represent the optical flow of the head and by applying the same COG approach as before the position of the head is defined. The resulting time series is denoted $(x^{\text{raw}}_{1,\text{head}}[k], x^{\text{raw}}_{2,\text{head}}[k])$.

If $k = 1$ (video just started), we set the head estimation to the COG, and if no head estimation can be calculated (e.g. due to missing movement), we retain the last one. As a final step, we smooth (low-pass filtering over time) the estimated head position values. The resulting time series is denoted $(x_{1,\text{head}}[k], x_{2,\text{head}}[k])$.

The center of gravity is predicted by linear extrapolation (quadratic functions do not work!) according to

$$(\Delta x_{1,\text{COG,p}}[k + \Delta k], \Delta x_{2,\text{COG,p}}[k + \Delta k]) = (a_0 + a_1 \Delta k, b_0 + b_1 \Delta k), \tag{6}$$

a_i, b_i are estimated based on the last five values of $(x_{1,\text{COG}}[k-j], x_{2,\text{COG}}[k-j], j = 0, ..., 4)$ (ordinary least squares). Predictions are very sensitive, therefore we low-pass filter the found $(x_{1,\text{COG,p}}[k + \Delta k], x_{2,\text{COG,p}}[k + \Delta k])$ values over time.

The segmented worm is filtered with a Gaussian convolution filter (to remove noisy edges), and the segment is thinned to a line (skeletonization, see Fig 3g).

To predict an arbitrary position within the worm (which can be identified by a mouse click later-on), we introduce a normalized coordinate system along the skeleton line of the worm (0: head, 1: tail). A click point $(x_{1,c}[k], x_{2,c}[k])$ at time sample k can then be transformed into a percentage value $s[k] \in [0, 1]$ defining the relative position of the click within the worm. For past time samples, the Cartesian coordinates of the same s-value are calculated and the velocity of the click point along the line coordinate system is calculated and filtered.

Assuming that the shape of the worm stays roughly the same[2], the velocity along the shape is used to predict where the click point will be located after Δk time samples. The predicted points are filtered as well. The resulting time series is denoted $(x_{1,c,p}[k], x_{2,c,p}[k])$.

Similarity measure

We compare the simulation outcome to the true image by the structural similarity measure that was introduced in [1]. This measure assesses the similarity between images

[2]The shape does not stay exactly the same, however this assumption simplifies processing and causes only small errors.

X and Y, based on the luminance l, the contrast c, and the structure st. Using μ as the brightness mean, σ^2 as the brightness variance, and σ_{xy} as the brightness co-variance, similarity is calculated according to

$$s_{xy} = \left[l(X,Y)^{\alpha} \cdot c(X,Y)^{\beta} \cdot st(X,Y)^{\gamma} \right] \tag{7}$$

where

$$l(X,Y) = \frac{2\mu_x\mu_y + c_1}{\mu_x^2 + \mu_y^2 + c_1},$$

$$c(X,Y) = \frac{2\sigma_x\sigma_y + c_2}{\sigma_x^2 + \sigma_y^2 + c_2},$$

$$st(X,Y) = \frac{\sigma_{xy} + c_3}{\sigma_x\sigma_y + c_3}.$$

α, β, and γ are weights of the luminance, contrast, and structure respectively. Within this paper we assumed an equal effect of the three terms, thus $\alpha = \beta = \gamma = 1$.

c_1, c_2, and c_3 are small real non-negative variables used to stabilize the division when the denominator is small. The default values of these constants are $c_1 = (0.01 * L)^2$, $c_2 = (0.03 * L)^2$, and $c_3 = c_2/2$ with L being the dynamic range of the images. $S(X,Y)$ takes values between 0 and 1, where 0 corresponds to no similarity, while 1 represents a complete similarity between the images.

Quality of MR imaging in dependence of prediction horizon

Fig. S1 shows the statistical assessment of the effect of the prediction horizon on the quality of the MR imaging. The data in this figure is taken from the simulation results for four slices along the worm and from eight videos of different worms. The figure shows that the prediction algorithm works quite well for all worms, and the results exhibit a high degree of resemblance. Moreover, and as expected, the results demonstrate how the accuracy of prediction and thus the MRI quality decays with the increased prediction horizon.

References

1. Wang Z, Bovik AC, Sheikh HR, Simoncelli EP. Image quality assessment: from error visibility to structural similarity. IEEE Transactions on Image Processing. 2004;13(4):600–612.
