# Supplementary figures and images for: Motion prediction enables simulated MR-imaging of freely moving model organisms
Source: PLoS Comput Biol. 2019 Dec 19;15(12):e1006997. doi: 10.1371/journal.pcbi.1006997 (PMC6941817; doi:10.1371/journal.pcbi.1006997)

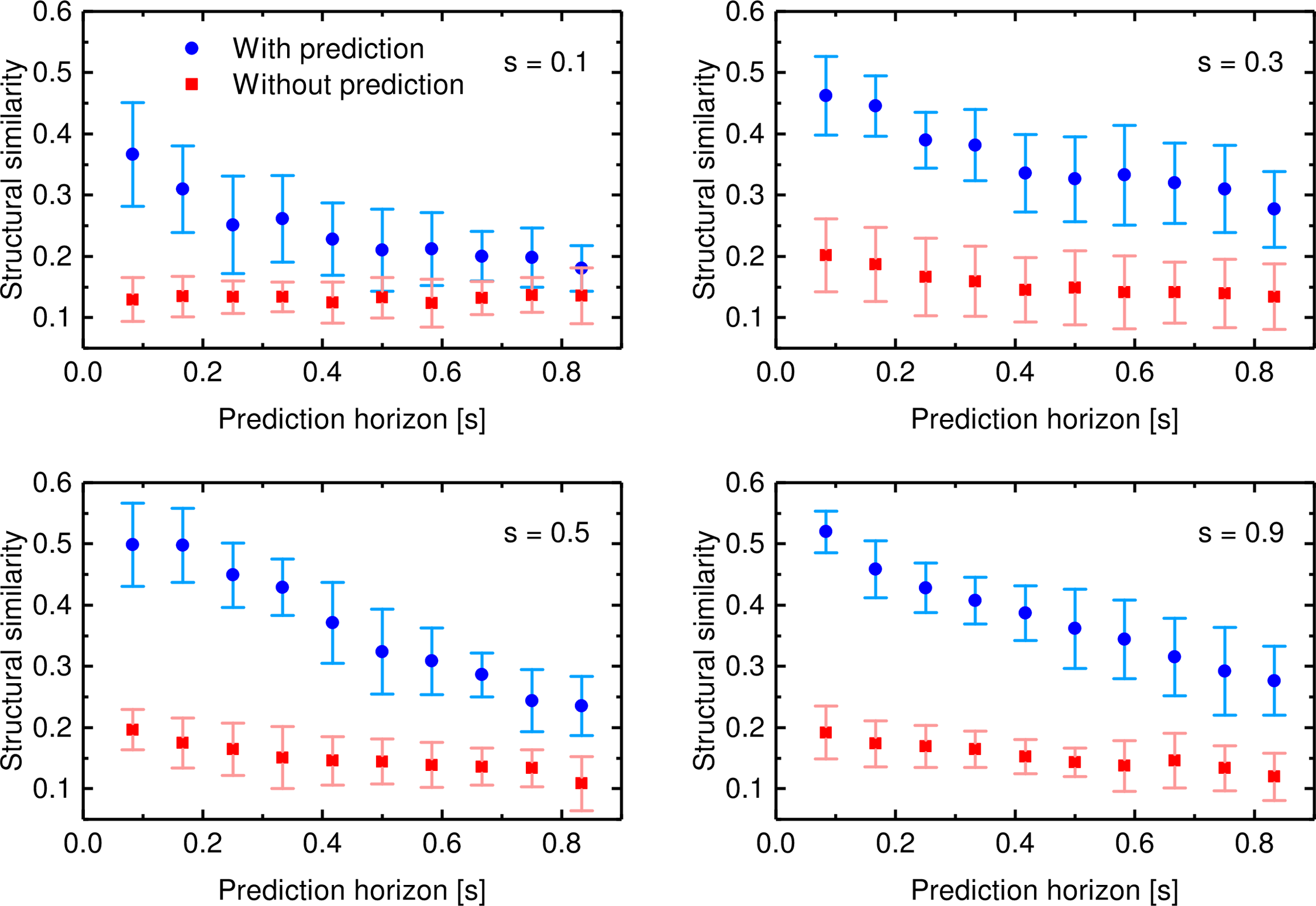

Supplement: S1 Fig — (TIF) [file pcbi.1006997.s002.tif]
